# Screening, identification, and low‐energy ion modified breeding of a yeast strain producing high level of 3‐hydroxypropionic acid

**DOI:** 10.1002/mbo3.956

**Published:** 2019-10-20

**Authors:** Wen Li, Tao Wang, Yuwei Dong, Tongxiang Li

**Affiliations:** ^1^ Jiangsu Key Construction Laboratory of Food Resource Development and Quality Safe Xuzhou University of Technology Xuzhou P. R. China

**Keywords:** 3‐Hydroxypropionic acid (3HP), low‐energy ion, modification, mutagenesis, yeast

## Abstract

3‐Hydroxypropionic acid (3HP) is an important platform chemical with a wide range of applications. The biological preparation of this compound is safe and low cost. In this study, orchard soil and human waste were used as raw materials to screen microbial strains that could produce 3HP in selective medium containing varying amounts of propionic acid. A yeast strain that can use propionic acid as substrate and produce 48.96 g/L 3HP was screened. Morphological observation, physiological and biochemical identification, and 26s rDNA sequencing identified the IS451 strain as *Debaryomyces hansenii*. The low‐energy ion N^+^, with the energy of 10 keV and a dose of 70 × 2.6 × 10^13^ ions/cm^2^, was implanted into the IS451 strain. The mutant strain WT39, whose 3HP titer reached 62.42 g/L, was obtained. The strain exhibited genetic stability and tolerance to high concentrations of propionic acid and was considered to have broad application prospects.

## Graphical abstract

The yeast *Debaryomyces hansenii* strain IS451 that can use propionic acid as substrate and produce 48.96 g/L of 3‐hydroxypropionic acid (3HP) was screened. The low‐energy ion N+ was implanted into the IS451 strain. The mutant strain WT39 whose 3HP titer reached 62.42 g/L was obtained. The strain exhibited tolerance to high concentrations of propionic acid and was considered to have broad application prospects.

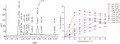

## 1 INTRODUCTION

The development of bio‐based resources has become an important strategy to improve the security of energy resources, reduce green house gas emissions, and address climate change (Kumar, Ashok, & Park, 2013). 3‐Hydroxypropionic acid (3HP) is an significant chemical intermediate that contains two functional groups, namely, hydroxyl and carboxyl (Ko, Ashok, Zhou, Kumar, & Park, 2012). 3HP, as the precursor of many optically active substances and an important platform chemical, directly affects the production of many high value‐added chemicals. 3HP can be used to produce malonic acid, 1,3‐propanediol, succinic acid, special polyester, and acrylicacid (Ashok et al., 2013; Henry, Broadbelt, & Hatzimanikatis, 2010; Zheng, Zhang, Zhang, & Chen, 2004). 3HP can also be used to generate various critical fine chemical products. Currently, 3HP is a chemical product with high potential for development by the US Department of Energy (Jiang, Meng, & Xian, 2009). 3HP is traditionally prepared through chemical synthesis. For example, it is produced by the reaction between adjacent halogenated glycol and potassium cyanide through hydrolysis or Reformatsky reaction (Ishida & Ueno, 2000). Chemical synthesis for 3HP production is difficult and expensive. The corresponding separation and purification procedures are also complicated.

Biological methods can effectively avoid the limitations of chemical synthesis and possess low cost, mild conditions, and other advantages. Up to now, several organisms have been reported to produce 3‐HP from at least six compounds: glycerol (Ashok, Raj, Rathnasingh, & Park, 2011; Li, Wang, Ge, & Tian, 2016; Lim, Noh, Jeong, Park, & Jung, 2016; Raj, Rathnasingh, Jo, & Park, 2008; Su, Li, Ge, & Tian, 2015; Zhou, Catherine, Rathnasingh, Somasundar, & Park, 2013), malonyl‐CoA (Chen, Bao, Kim, Siewers, & Nielsen, 2014; Rathnasingh et al., 2012), acrylic acid (Lee, Park, Park, Cho, & Rhee, 2009), β‐alanine (Borodina et al., 2015; Song, Kim, Cho, & Lee, 2016), 3‐hydroxypropionitrile (Yu et al., 2016), or propionic acid (Luo et al., 2016). Among those, the biological pathways from malonyl‐CoA and glycerol for 3‐HP production are extensively reported.

Moreover, the genetically engineered bacteria have been increasingly used to prepare 3HP, which has become one of the most notable research focus worldwide (Huang, Li, Shimizu, & Ye, 2011). Several studies lean on bacterial hosts such as *Escherichia coli* (Chu et al., 2015; Jung, Kang, Chu, Choi, & Cho, 2014; Kim, Kim, Park, & Seo, 2014; Kwak, Park, & Seo, 2013; Raj et al., 2008) and *Klebsiella pneumonia* (Ashok et al., 2011; Kumar, Sankaranarayanan, et al., 2013; Li et al., 2016). Genes encoding malonyl‐CoA reductase, acetyl‐CoA decarboxylase, biotin enzymes, and transhydrogenase were expressed in recombinant *Escherichia coli* BL21, which could produce 1.2 mmol/L 3HP by using glucose as substrate (Raj et al., 2008), the 3HP titers reached 71.9 g/L and 83.8 g/L in recombinant *E. coli* W3110 and *K. pneumoniae* DSM 2026 with glycerol as the carbon source (Chu et al., 2015; Li et al., 2016).

Construction of genetically engineered bacteria involves many genes and faces enormous challenges in actual production; such challenges include the stability of genes in a host cell, enzyme activity, flow regulation of cellular metabolism, and other factors. Few studies have reported on the use of natural bacteria to generate 3HP, the natural producers include both prokaryotes and eukaryotes. However, the 3HP titer is low. Wild strains that can be used to produce 3HP mainly include lactic acid bacteria (Garai‐lbabe et al., 2008; Krauter, Willke, & Vorlop, 2012; Sobolov & Smiley, 1960; Talarico, Casas, Chung, & Dobrogosz, 1988), *Metallosphaera sedula* (Berg, Kockelkorn, Buckel & Fuchs, 2007), *Rhodococcus erythropolis* (Lee et al., 2009), *Hansenula miso* (Harada & Hirabayashi, 1968), *Fusarium merismoides* (Miyoshi & Harada, 1974), *Candida rugosa* (Hasegawa, Ogura, Kanema, & Kawaharada, 1982), *Byssochlamys sp*. (Takamizawa, Horitsu, Ichikawa, & Kawai, 1993), *Saccharomyces kluyveri* (Andersen et al., 2008) and *Meyerozyma guilliermondii* (Zhang et al., 2017). In a rich repository of microorganisms, many 3HP‐producing microbes have not been discovered yet because of their diversity in the natural environment. Thus, microbial strains that can generate 3HP must be further investigated. Screening and breeding of new 3HP‐producing strains can enrich the species of 3HP‐producing microbes, provide the basis for the research and application of biochemical industry and biological fermentation, moreover, which can provide new resources for the construction of 3HP efficient engineering bacteria, benefit human beings, and solve the problems of energy security.

Organisms can be modified after the implantation of low‐energy ion, considering the effects of energy deposition, mass deposition, and charge exchange. The low‐energy ion is characterized by low damage, high mutation rate, and wide mutational spectrum (Yu, 2000). In 1995, scholars found the biological effects of ion implantation; since then, this ion has been widely used for breeding high‐yield industrial microbial strains and crops and for research on mammal radiation biology and other fields. Incorporating this ion leads to good economic benefits of a plurality of microorganisms and crop species (Feng, Yu, & Chu, 2006). *Cordyceps militaris* strains containing abundant Selenium were obtained by ion implantation (Wang, Li, Chen, Li, & Zhao, 2014). Low‐energy ion can be applied to breed different kinds of bacteria that produce D(–)‐lactic acid, vitamin C, rifamycin, tannase, microbial lipid, and arachidonic acid, separately (Chen et al., 1998; Nie et al., 2013; Wang, Zhang, Zheng, & Wang, 2011; Xu, Bai, Wang, & He, 2010; Yu et al., 2012; Yu, Xu, Wang, & Yu, 1999).

This work aims to breed strains that can produce high titer of 3HP. In the proposed strategy, 3HP high‐producing strains were first screened from orchard soil and feces samples. The strain was then identified based on morphological observation, physiological and biochemical identification, and 26s rDNA sequencing. The 3HP titer was finally increased by implanting low‐energy ion into the strain. Results will provide a basis for developing 3HP engineered bacteria, and for industrial production of 3HP by immobilized bacteria.

## 2 MATERIALS AND METHODS

### 2.1 Materials

Soil samples were collected from orchard and duck ring. Human feces samples were obtained from healthy adults. 3HP was purchased from HaoRui Chemical Inc. Methanol used for high‐performance liquid chromatography (HPLC) and propionic acid were acquired from Sinopharm Chemical Reagent Co., Ltd. All of the other reagents were of analytical grade.

### 2.2 Medium

Four kinds of media were prepared as follows: slant medium (g/L): glucose 20.00, yeast extraction 10.00, peptone 20.00, agar 20.00; seed medium (g/L): glucose 20.00, yeast extraction 10.00, (NH_4_)_2_SO_4_ 10.00, KH_2_PO_4_5.00, K_2_HPO_4_ 2.00, MgSO_4_.7 H_2_O 1.00, FeSO_4_.7 H_2_O 0.03, TES 5 ml (TES was a mixture of trace element solution and was composed of (g/L): HCl 3.65, H_3_BO_4_ 0.30, CuCl·6H_2_O 0.20, ZnSO_4_.7H_2_O 0.10, MnSO_4_ 0.03, NaMoO_4_.2H_2_O 0.03, NiCl_2_.6H_2_O 0.02, CuSO_4_.5H_2_O 0.01); fermentation medium (g/L): glucose 30.00, yeast extraction 10.00, KH_2_PO_4_ 5.00, K_2_HPO_4_ 2.00, (NH_4_)_2_SO_4_ 10.00, MgSO_4_.7H_2_O 1.00, FeSO_4_.7 H_2_O 0.03, propionic acid 10.00, TES 5 ml, pH 6.0; and selection medium (g/L): glucose 30.00, yeast extraction 10.00, (NH_4_)_2_SO_4_ 10.00, KH_2_PO_4_ 5.00, K_2_HPO_4_ 2.00, MgSO_4_.7 H_2_O 1.00, FeSO_4_.7 H_2_O 0.03, TES 5 ml, propionic acid 4–20, agar 20.00.

### 2.3 Screening of 3HP‐producing strains

Samples collected from orchard soil and human feces were soaked in sterile saline and allowed to stand for 12 hr. The suspension was treated by 10‐fold serial dilutions with sterile saline. Briefly, 100 µl of the aliquot for each concentration was coated on selection medium with 0.4% propionic acid and incubated at 30°C for 72 hr. Single colonies growing well were selected, purified, and placed on selection medium with 1% propionic acid. The strains were incubated at 120 r/min for 20 hr at 30°C in 50 ml of the seed medium. The strains were then transferred with 4% (v/v) inoculate to a 250‐ml Erlenmeyer flask containing 50 ml of the fermentation medium and fermented at 30°C for 48 hr. Each flask fermentation was performed in triplicate. After sampling and centrifugation at 8,000 r/min for 10 min, the supernatant was used for 3HP determination.

### 2.4 Determination of 3HP

3HP concentration was analyzed by HPLC with an Ecosil C18 Column (250 mm × 4.6 mm, 5 μm, Diamonsil). The sample (20 μl) was injected and monitored at 210 nm wavelength by using a DAD detector (SPD‐10AVP, Shimadzu). The elution solvent system was composed of 3% methanol (pH was adjusted to 2.0 with H_3_PO_4_), and a flow rate of 0.8 ml/min was used. The temperature of the column oven was set at 35°C. 3HP was used as a standard sample to draw a standard curve. The calibration curve was obtained based on the 3HP standard.

### 2.5 Identification of 3HP‐producing strains

#### 2.5.1 Morphological observation of strain

The cultural and morphological characteristics of the selected strain IS451 were determined.

#### 2.5.2 Identification of biochemical characteristics

Biochemical characteristics were determined according to the method of Barnett, Payne, and Yarrow (Barnett, Payne, & Yarrow, 2000).

#### 2.5.3 Molecular identification

The 26S rDNA sequence of the strain IS451 was amplified using the forward primer 5′‐CAGAGTTTGATCCTGGCT‐3′ and the reverse primer 5′‐AGGAGGTGATCCAGCCGCA‐3′, which were designed according to the conservative region of the 26S rDNA D1/D2 sequences. The PCR products were sequenced by Sangon Biotech Co., Ltd. The sequences determined were submitted to the GenBank Database and aligned with the 26S rDNA D1/D2 domain sequences acquired from the data base by using the BLAST program. The taxonomic status of the strains was then determined.

### 2.6 Ion implantation and mutant selection

#### 2.6.1 Preparation of samples

A single colony of high‐producing 3HP IS451 strain was obtained and cultured in seed medium at 120 r/min for 12 hr. After centrifugation at 5,000 r/min for 2 min, cells were collected and resuspended in an equal volume of PBS (pH 7.0) buffer. Subsequently, 0.1 ml of the suspension with 10^1^ dilutions was spread as a single‐cell layer on a sterilized petri plate. The plate was desiccated by filtrated air on a clean bench before ion implantation.

#### 2.6.2 Ion implantation conditions

The implantation sources were produced by an ion beam bioengineering instrument (Patent No. ZL93103361.6, Yu, 2000, P.R.C.) devised by ASIPP (Institute of Plasma Physics, Chinese Academy of Sciences).

Vacuum degree injected into the target chamber was 10^–3^ Pa, and the beam flow was 1 mA. Ion implantation energy was 10 keV. The implantation dose ranged from 10 × 2.6 × 10^13^ ions/cm^2^ to 170 × 2.6 × 10^13^ ions/cm^2^, and the ion species was N^+^. The spores of the control group without N^+^ beam implantation were also placed in the target chamber to evaluate the vacuum effects on the mutation.

#### 2.6.3 Screening of mutant strains

The target samples subjected to ion implantation were immediately eluted with PBS. Subsequently, 0.1 ml of the washed sample was coated on a high‐concentration selection medium containing 1.5% propionic acid. The colonies growing on the plate were transferred to a slant medium, inoculated into the fermentation medium, and cultured for 3 days. The 3HP titer was examined to screen high‐producing mutant strains.

### 2.7 Genetic stability of the strain WT39

The high‐producing 3HP mutant strain was continuously passaged in the slant medium. The titer of 3HP produced was measured and compared with the original generation strains to determine the genetic stability of the mutant strain WT39.

### 2.8 Tolerance test of original and mutant strains to propionic acid

The strains were inoculated into fermentation medium with different concentrations of propionic acid (0, 5, 15, 25, and 35 g/L) and cultured at 30°C for 96 hr. The cells cultured at different fermentation time points were collected by centrifugation at 5,000 r/min for 2 min. The precipitates were freeze‐dried. The dry weight of the original and mutant strains was determined.

### 2.9 Statistical analysis

All tests were carried out in triplicate. Data were expressed as mean ± standard deviation (*SD*). Data analyses were performed with SPSS 14.0 (SPSS Inc.). One‐way analysis of variance (ANOVA) was conducted to determine significance differences. *p* < .05 was considered to be statistically significant.

## 3 RESULTS

### 3.1 Screening of 3HP‐producing strains

#### 3.1.1 Primary screening

After 48 hr of incubation, colonies that grew well on selection medium containing 0.4% propionic acid were selected and subjected to three consecutive steps to obtain pure colonies. A total of 418 strains selected were preserved in the slant medium for subsequent screening.

#### 3.1.2 Secondary screening

**Figure 1:**
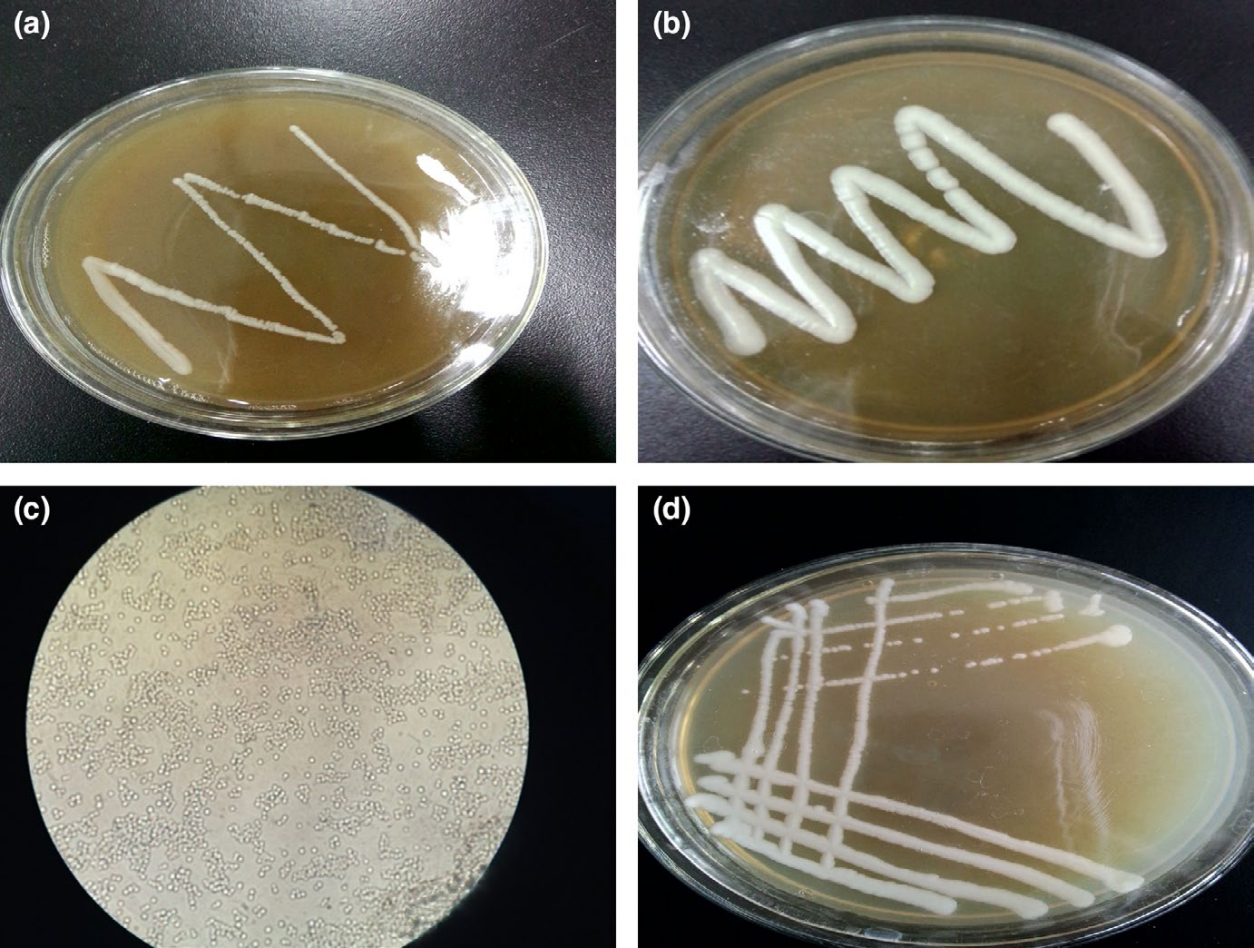
Morphology of the colonies of the strains IS141 (a), IS451 (b), single‐cell (400×) (c), and the single colony of the strain IS451 (d)

Strains obtained from the primary screening were inoculated into selective medium containing 1% propionate and incubated at 30°C for 48 hr. A total of 158 strains from the secondary screening were obtained. Compared with other 149 strains, IS153, IS258, IS269, IS375, IS424, IS433, IS466 showed better growth; IS141 and IS451 showed the best growth (Figure 1a,b; Table 1).

**Table 1:** Growth of strains on a selective medium containing 1% propionate

Strains	Growth	Strains	Growth
IS141	＋＋＋	IS424	＋＋
IS153	＋＋	IS433	＋＋
IS258	＋＋	IS451	＋＋＋
IS269	＋＋	IS466	＋＋
IS375	＋＋	Other strains	＋

Note

＋＋＋, grew best; ＋＋, grew better; ＋, grew.

**Figure 2:**
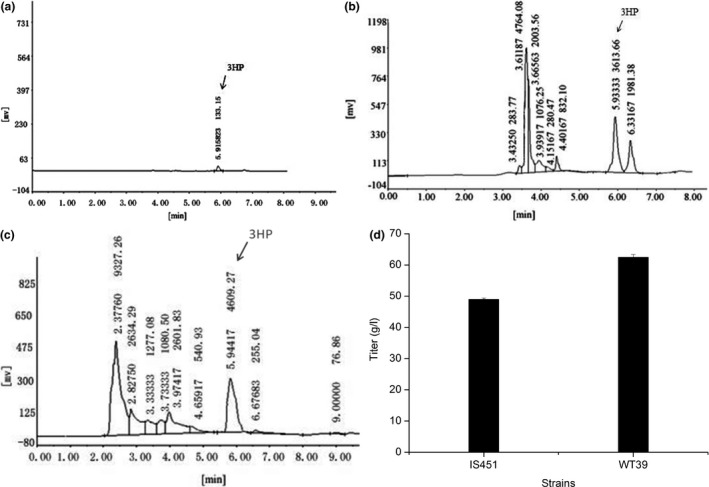
HPLC chromatograms of 3HP standard sample(4 g/L) (a), RT = 5.916 min, cultural supernatant of strain IS451 (b), RT = 5.933 min, WT39 (c), RT = 5.944 min. HP peak was indicated by an arrow, and 3HP production of strains IS451 and WT39 (d)

After being cultured in the fermentation medium, 158 isolates were tested for 3HP‐producing ability by HPLC determination. Among these strains, 18 isolates produced 3HP by using propionic acid as a substrate. Six isolates, namely, IS052, IS141, IS256, IS353, IS451, and IS456, produced higher 3HP titer of c. 6.89, 11.41, 4.69, 8.92, 48.96, and 7.24 g/L after 48 hr of fermentation, respectively. Strain IS451 produced the highest 3HP content. Furthermore, the same 3HP peak was found in the HPLC chromatogram of the 3HP standard sample and culture supernatant of the strain IS451 (Figure2a,b). Thus, strain IS451 was confirmed to produce 3HP and chosen as the 3HP‐producing strain in the following experiment.

### 3.2 Identification of strain IS451

#### 3.2.1 Morphological observation and physiological and biochemical identification

Microscopic observation results showed that the single‐cell of strain IS451 was elliptical or circular (Figure 1c). The colonies of the strain were circular, opaque, oyster white, convex, soft, and wet with the neat edge (Figure1d). The physiological and biochemical characteristics of the IS451 strain were shown in Table 2. Glucose, sucrose, maltose, lactose, and soluble starch were assimilated by strain IS451 as carbon sources. Ammonium sulfate, ammonium nitrate, and potassium nitrate were utilized as nitrogen sources, whereas urea was not utilized. The optimal pH range of the strain was 4.0–6.0, and the optimum growth temperature was 30°C. Thus, strain IS451 was considered to be yeast based on its morphological and biochemical characteristics.

**Table 2:** Physiological and biochemical characteristics of strain IS451

Item	Result	Item	Result
Glucose	＋	Ammonium sulfate	＋
Sucrose	＋	Ammonium nitrate	＋
Lactose	＋	Urea	−
Soluble starch	＋	Optimum pHrange	4.0–6.0
Maltose	＋	Optimum temperature	30°C
Potassium nitrate	＋		

Note

+, positive or could grow; −, negative or could not grow.

#### 3.2.2 26S rDNA sequencing of strain IS451

The 26S rDNA sequence of strain IS451 was 579 bp long and showed 99% homology with that of *Debaryomyces hansenii*strains WHCX and QD9.1. According to the analysis of morphological, physiological, and biochemical characteristics and sequence alignment results, strain IS451 was identified to be *D. hansenii*. The *D. hansenii* IS451 strain was deposited in NCBI Genbank under the accession number of KY264052.

### 3.3 Improvement of 3HP production of *D. hansenii* IS451 by low‐energy ion implantation

#### 3.3.1 Ion implantation

**Figure 3:**
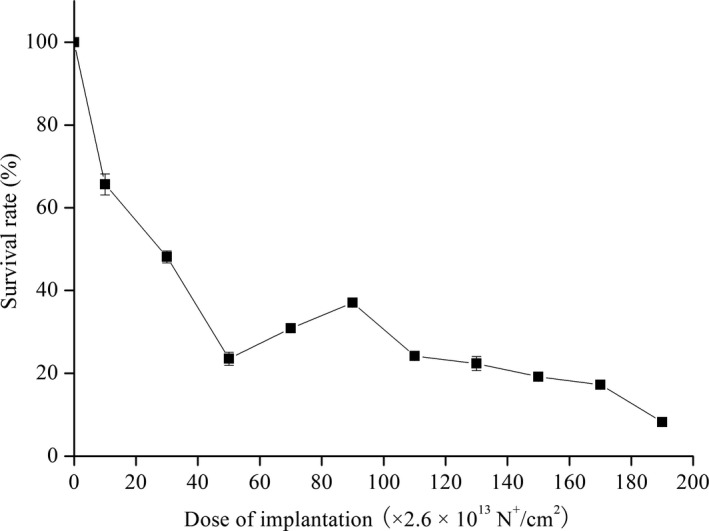
Surviving curve of strain IS451 after N^+^ ion implantation. Data are expressed as mean ± *SD* from triplicate experiments

The damage effects of different low‐energy ions on organisms and the sensitive degree presented were different. For the three common ions of H^+^, N^+^, and Ar^+^, N^+^ was confirmed to cause the highest mutagenic efficiency (Tang et al., 2007). Therefore, 10 keV of N^+^ ions was selected and implanted into *D. hansenii* IS451. The surviving rate curve ignoring the effect of vacuum was shown in Figure 3. The survival rate of strains without N^+^ implantation was set as 100% with increasing N^+^ dose. The survival rate of the strain first decreased, then increased, and finally decreased again; this trend is consistent with “saddle‐shaped” dose‐response curve. This curve was considered to be caused by the damaging effect of energy and momentum and the protective effect and comprehensive stimulus of the quality and charge. At a dose of 0–50 × 2.6 × 10^13^ ions/cm^2^, the survival rate decreased rapidly, and the survival rate increased slowly with increasing dose. When the dose reached 90 × 2.6 × 10^13^ ions/cm^2^, the survival rate began to decrease. The experimental condition corresponding to 20%–30% survival rate would be chosen for breeding microorganisms, because under such condition, the positive mutation rate would be the highest (Zhu & Wang, 1994). Thus, in the present study, the optimum dose of N^+^ implantation was 70 × 2.6 × 10^13^ ions/cm^2^. This dose could not only guarantee the survival rate but also show a certain mutation rate.

#### 3.3.2 Screening mutants with high 3HP production

Numerous implantation mutation experiments were carried out using low‐energy ion N^+^ with the energy of 10 keV and a dose of 70 × 2.6 × 10^13^ ions/cm^2^. The mutant strains were screened on selection medium with 20 g/L propionic acid. Colonies with high H/D ratio were selected and fermented for 48 hr. Strain WT39 produced the highest 3HP content of 62.42 g/L after 48 hr of fermentation, the HPLC chromatogram of the fermentation broth for WT39 was shown in Figure 2c; Ion implantation resulted in the significant increases in 3HP titer and productivity, the 3HP titer of WT39 (62.42 g/L) increased by 27.5% compared with that of the original strain IS451 (48.96 g/L; Figure 2d), and the productivity increased from 1.02 g/(L·h) to 1.30 g/(L·h).

### 3.4 Hereditary stability test

The 3HP high‐producing strain WT39 was continuously passaged for 10 times. 3HP production was determined and compared with the first generation. The results were shown in Table 3. The amount of 3HP produced was not significantly different (*p* < .05) among different generations of strain WT39. Thus, WT39 exhibited good genetic stability and was suitable for industry production.

**Table 3:** Genetic stability test

Generation number	3HP (g/L)
1	62.42 ± 1.00^a^
2	62.53 ± 0.76^a^
3	61.66 ± 0.59^a^
4	61.38 ± 1.15^a^
5	61.51 ± 1.67^a^
6	61.29 ± 0.90^a^
7	61.69 ± 1.26^a^
8	61.61 ± 1.21^a^
9	60.55 ± 0.72^a^
10	61.34 ± 0.93^a^

Note

Data are expressed as mean ± *SD* from triplicate experiments. Different superscripts within the same column indicate a significant difference (*p* < .05).

### 3.5 Tolerance of original and mutant strains to propionic acid

**Figure 4:**
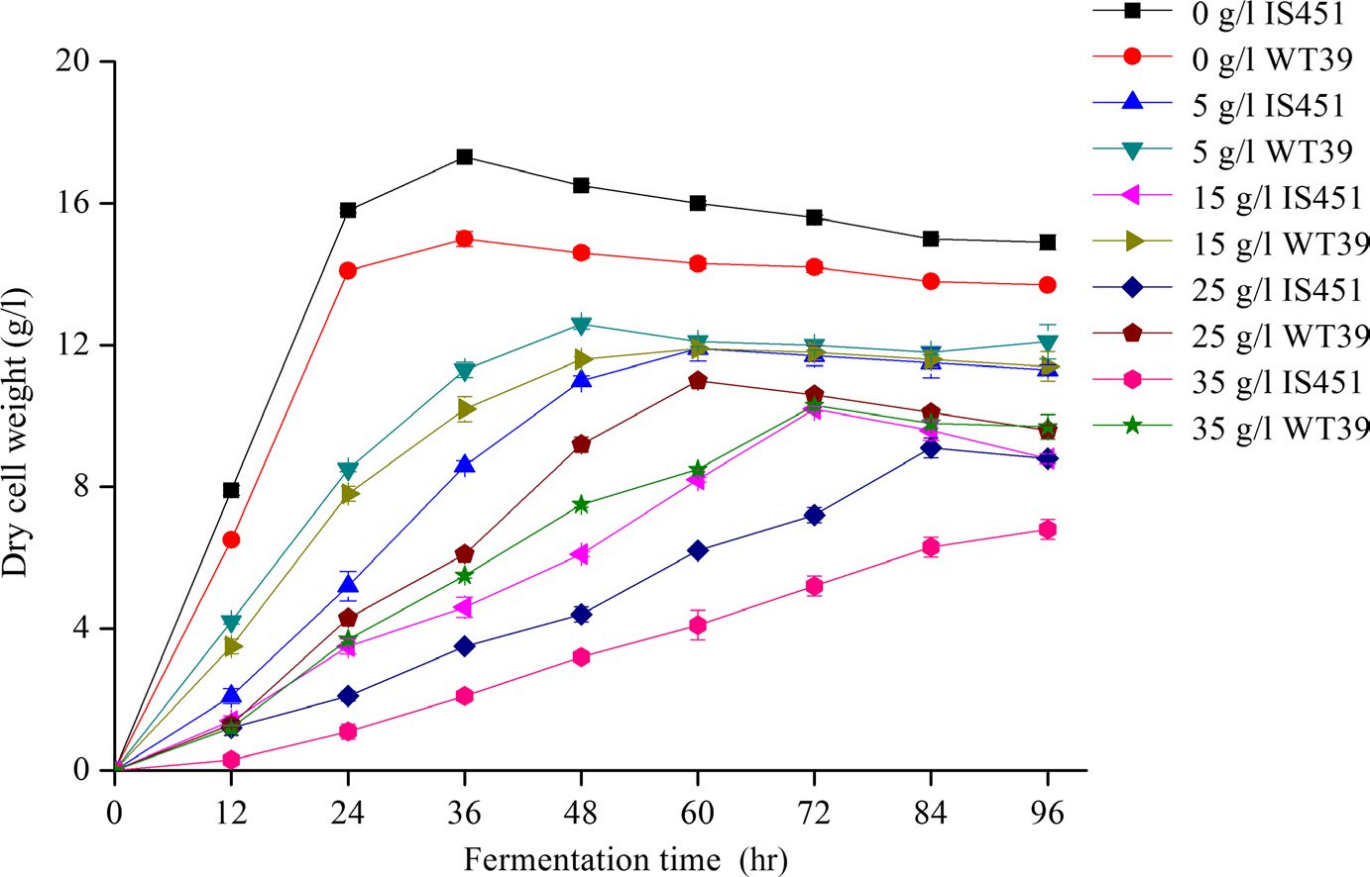
Propionic acid tolerance of strains IS451 and WT39. Data are expressed as mean ± *SD* from triplicate experiments

Propionic acid, as a kind of organic acid, can inhibit the growth of microorganisms at high concentrations. As the substrate of 3HP, propionic acid tolerance of strain is the crux of fermentation and can be used as the main indicator of 3HP high‐producing strains. With increasing concentration of propionic acid, the stationary phase of the original and mutant strains lagged (Figure 4). This finding indicated that high concentrations of propionic acid inhibited the growth of the strains. In the absence of propionic acid, the two strains grew well and reached the stationary phase after 24 hr. The original strains grew better than the mutant strain. However, the mutant strain grew better than the original strain with increasing propionic acid concentration. Therefore, the mutant strain showed improved propionic acid tolerance.

## 4 DISCUSSION

Bio‐synthetic production of 3HP can avoid the limitations of chemical synthesis. In this study, the 3HP high‐producing strain IS451 was obtained from the orchard soil. The strain IS451 was identified as *D. hansenii* by morphological observation, physiological, biochemical identification, and through analysis of the D1/D2 sequence in 26S rDNA. The amount of 3HP produced by the strain was 48.96 g/L. Li and Pei (2010) screened the microbial strain *K. terrigena*, which could use glycerol as the sole carbon source to produce 10 g/L 3HP from the natural environment. Fan, Fang, Zhu, and Zhu (2012) obtained a strain which could utilize propionic acid to produce 4.78 g/L 3HP by fermentation. The strain was identified as *Candida* sp. by physiological and biochemical identification and 18S rDNA sequence analysis. To our knowledge, the present study is the first to demonstrate the production of 3HP by *D. hansenii*, which enriched the microbe resources for 3HP production.

The low‐energy ion N^+^, with the energy of 10 keV and a dose of 70 × 2.6 × 10^13^ ions/cm^2^, was implanted into IS451 strain. The mutant strain WT39 produced 62.42 g/L 3HP, which increased by 27.5% compared with that of the original strain. The strain WT39 also exhibited good hereditary stability and showed tolerance to high concentrations of propionic acid compared with the original strain IS451. As far as we know, the amount of 3HP produced by strain WT39 was higher than that of the other wild and mutant 3HP‐producing strains previously reported, except for the genetically engineered bacteria. Although the 3HP titer by WT39 was lower than some of the genetically engineered bacteria such as *E. coli* W3110 and *K. pneumonia* DSM 2026, the productivity of WT39 was higher than that of *K. pneumonia* DSM 2026, and also, *E. coli* W3110 and *K. pneumonia* DSM 2026 produce 3HP by glycerol pathway (Chu et al., 2015; Li et al., 2016), while WT39 via the propionyl‐CoA pathway. In addition, there were many disadvantages when using genetically engineered bacteria, such as the instability of genes in a host cell, enzyme activity, flow regulation of metabolism. When compared to chemical synthesis for 3HP production, production process by WT39 is easier and economically viable. Therefore, the strain WT39 exhibits a broad application prospect in the industry. The interaction between the low‐energy ions and the biological materials is more complicated than that of γ‐ray or X‐ray radiation. Ion beam implantation, as a new mutation method, has been characterized by wider mutation spectra and higher mutation frequence (Ge, Gu, Zhou, & Yao, 2004). Ion beam genetic modification of microbes and plants is now a well‐established mutation breeding technique, by which many new microbe strains and crop varieties have been bred and are currently used in practice (Feng et al., 2006). In the studies of microbial breeding, ion energy was 10 keV, and the dose was usually 40 × 2.6 × 10^13^ – 100 × 2.6 × 10^13^ ions/cm^2^. The survival rate‐dose of relationship of IS451 did not follow exponential law, but exhibited “down–up–down” pattern, which was not same with the traditional mutagen irradiation, such as γ‐ray and UV, with a low‐energy ion beam showed a characteristic curve like “saddle”, and there were the abnormal radiation damages induced by ion beam. This saddle type curve was also found by Tang et al. (2007) and Li, Zhu, Gu, Liu, and Wang (2011). According to the experimental results, we may deduce that there may be a new repair mechanism in IS451 during ion beam exposure, which has not been discovered and distinguished from the repair of recombination, excision and error‐prone repair. Although the mechanism of ion beam mutation is not illuminated, this research suggests that low‐energy ion beam irradiation is a valuable mutagensource. It could be widely applied to the microbe breeding and could improve the selection efficiency. Further studies must be performed to determine the molecular mechanisms through which N^+^ implantation improves the 3HP production ability of the strain.

## ETHICS STATEMENT

None required.

## CONFLICT OF INTERESTS

None declared.

## AUTHORS CONTRIBUTION

Wen Li: screened the 3HP‐producing strains, observed the strains, identified the biochemical characteristics**,** contributed to experimental design, and wrote the manuscript. Tao Wang: implanted ion beam into the strain, performed the HPLC, and contributed to experimental design, and wrote the manuscript. Yuwei Dong: extracted 26S rDNA and compared the sequence to other sequences available. Tongxiang Li: conducted the genetic stability and propionic acid tolerance experiments.

## Data Availability

All data are provided in the results of the manuscript. The 26S rDNA sequence of strain IS451 is located at https://www.ncbi.nlm.nih.gov/nuccore/ under the accession number KY264052.

## References

[mbo3956-bib-0001] Andersen, G. , Björnberg, O. , Polakova, S. , Pynyaha, Y. , Rasmussen, A. , Møller, K. , … Piškur, J. (2008). A second pathway to degrade pyrimidine nucleic acid precursors in eukaryotes. Journal of Molecular Biology, 380(4), 656–666. 10.1016/j.jmb.2008.05.029 18550080

[mbo3956-bib-0002] Ashok, S. , Raj, S. M. , Rathnasingh, C. , & Park, S. (2011). Development of recombinant *Klebsiella pneumoniae* ∆dhaT strain for the coproduction of 3‐hydroxypropionic acid and 1, 3‐propanediol from glycerol. Applied Microbiology & Biotechnology, 90(4), 1253–1265. 10.1007/s00253-011-3148-z 21336929

[mbo3956-bib-0003] Ashok, S. , Sankaranarayanan, M. , Ko, Y. , Jae, K. E. , Ainala, S. K. , Kumar, V. , … Park, S. (2013). Production of 3‐hydroxypropionic acid from glycerol by recombinant *klebsiella pneumoniae*, Δ dhat Δ yqhd, which can produce vitamin B12 naturally. Biotechnology & Bioengineering, 110(2), 511–524. 10.1002/bit.24726 22952017

[mbo3956-bib-0004] Barnett, J. A. , Payne, R. W. , & Yarrow, D. (2000). Yeasts: Characteristics and identification. Cambridge, UK: C U P 10.1046/j.1525-1470.2001.1862020a.x

[mbo3956-bib-0005] Berg, I. A. , Kockelkorn, D. , Buckel, W. , & Fuchs, G. A. (2007). 3-hydroxypropionate/4-hydroxybutyrateautotrophic carbon dioxide assimilation pathway in Archaea. Science, 318(5857), 1782–1786. 10.1126/science.1149976 18079405

[mbo3956-bib-0006] Borodina, I. , Kildegaard, K. R. , Jensen, N. B. , Blicher, T. H. , Maury, J. , Sherstyk, S. , … Nielsen, J. (2015). Establishing a synthetic pathway for high‐level production of 3‐hydroxypropionic acid in*Saccharomyces cerevisiae* via β‐alanine. Metabolic Engineering, 27, 57–64. 10.1016/j.ymben.2014.10.003 25447643

[mbo3956-bib-0007] Chen, Y. , Bao, J. C. , Kim, I. K. , Siewers, V. , & Nielsen, J. (2014). Coupled incremental precursor and co‐factor supply improves 3‐hydroxypropionic acid production in *Saccharomyces cerevisiae* . Metabolic Engineering, 22, 104–109. 10.1016/j.ymben.2014.01.005 24502850

[mbo3956-bib-0008] Chen, Y. , Lin, Z. , Zou, Z. , Zhang, F. , Liu, D. , Liu, X. , Huang, B. (1998). High yield antibiotic producing mutants of Streptomyces erythreus induced by low energy ion implantation. Nuclear Instruments & Methods in Physics Research, 140(3‐4), 341–348. 10.1016/S0168-583X(98)00015-9

[mbo3956-bib-0009] Chu, H. S. , Kim, Y. S. , Lee, C. M. , Lee, J. H. , Jung, W. S. , Ahn, J. H. , … Cho, K. M. (2015). Metabolic engineering of 3‐hydroxypropionic acid biosynthesis in *Escherichia coli* . Biotechnology and Bioengineering, 112(2), 356–364. 10.1002/bit.25444 25163985

[mbo3956-bib-0011] Fan, J. , Fang, H. , Zhu, G. , & Zhu, G. (2012). Isolation, identification and mutation breeding of 3‐hydroxypropionic acid high production strain. Microbiology China, 39, 1355–1362.

[mbo3956-bib-0012] Feng, H. , Yu, Z. , & Chu, P. K. (2006). Ion implantation of organisms. Materials Science Engineering R, 54(3–4), 49–120. 10.1016/j.mser.2006.11.001

[mbo3956-bib-0013] Garai‐lbabe, G. , Ibarburu, I. , Berregi, A. , Claisse, O. , Lonvaud‐Funel, A. , Irastorza, A. , & Dueñas, M. T. (2008). Glycerol metabolism and bitterness producing lactic acid bacteria in cider making. International Journal of Food Microbiology, 121(3), 253–261. 10.1016/j.ijfoodmicro.2007.11.004 18180066

[mbo3956-bib-0014] Ge, C. , Gu, S. , Zhou, X. , & Yao, J. (2004). Breeding of L(+)‐lactic acid producing strain by low‐energy ion implantation. Journal of Microbiology and Biotechnology, 14(2), 363–365.

[mbo3956-bib-0015] Harada, T. , & Hirabayashi, T. (1968). Utilization of alcohols by *Hansenula miso* . Agricultural and Biological Chemistry, 32(9), 1175–1180. 10.1080/00021369.1968.10859202

[mbo3956-bib-0016] Hasegawa, J. , Ogura, M. , Kanema, H. , & Kawaharada, H. (1982). Production of β‐hydroxypropionic acid from propionic acid by a *Candida rugosa* mutant unable to assimilate propionic acid. Journal of Fermentation Technology, 60, 591–594.

[mbo3956-bib-0017] Henry, C. S. , Broadbelt, L. J. , & Hatzimanikatis, V. (2010). Discovery and analysis of novel metabolic pathways for the biosynthesis of industrial chemicals: 3‐hydroxypropanoate. Biotechnology & Bioengineering, 106(3), 462–473. 10.1002/bit.22673 20091733

[mbo3956-bib-0018] Huang, Y. , Li, Z. , Shimizu, K. , & Ye, Q. (2011). Simultaneous production of 3‐hydroxypropionic acid and 1,3‐propanediol from glycerol by a recombinant strain of *Klebsiella pneumoniae* . Bioresource Technology, 103(1), 351–359. 10.1016/j.biortech.2011.10.022 22055092

[mbo3956-bib-0019] Ishida, H. , & Ueno, E. (2000). Manufacture of 3‐hydroxypropionic acid with good selectivity and yield. JP 2000159724 A2 2000, 1.

[mbo3956-bib-0020] Jiang, X. , Meng, X. , & Xian, M. (2009). Biosynthetic pathways for 3‐hydroxypropionic acid production. Applied Microbiology and Biotechnology, 82(6), 995–1003. 10.1007/s00253-009-1898-7 19221732

[mbo3956-bib-0021] Jung, W. S. , Kang, J. H. , Chu, H. S. , Choi, I. S. , & Cho, K. M. (2014). Elevated production of 3‐hydroxypropionic acid by metabolic engineering of the glycerol metabolism in *Escherichia coli* . Metabolic Engineering, 23, 116–122. 10.1016/j.ymben.2014.03.001 24650754

[mbo3956-bib-0022] Kim, K. , Kim, S. K. , Park, Y. C. , & Seo, J. H. (2014). Enhanced production of 3‐hydroxypropionicacid from glycerol by modulation of glycerol metabolism in recombinant *Escherichia coli* . Bioresource Technology, 156, 170–175. 10.1016/j.biortech.2014.01.009 24502915

[mbo3956-bib-0023] Ko, Y. , Ashok, S. , Zhou, S. , Kumar, V. , & Park, S. (2012). Aldehyde dehydrogenase activity is important to the production of 3‐hydroxypropionic acid from glycerol by recombinant *Klebsiella pneumoniae* . Process Biochemistry, 47(7), 1135–1143. 10.1016/j.procbio.2012.04.007

[mbo3956-bib-0024] Krauter, H. , Willke, T. , & Vorlop, K. D. (2012). Production of high amounts of 3‐hydroxypropionaldehyde from glycerol by *Lactobacillus reuteri* with strongly increased biocatalyst lifetime and productivity. Nature Biotechnology, 29(2), 211–217. 10.1016/j.nbt.2011.06.015 21729774

[mbo3956-bib-0025] Kumar, V. , Ashok, S. , & Park, S. (2013). Recent advances in biological production of 3‐hydroxypropionic acid. Biotechnology Advances, 31(6), 945–961. 10.1016/j.biotechadv.2013.02.008 23473969

[mbo3956-bib-0026] Kumar, V. , Sankaranarayanan, M. , Durgapal, M. , Zhou, S. , Ko, Y. , Ashok, S. , … Park, S. (2013). Simultaneous production of 3‐hydroxypropionic acid and 1,3‐propanediol from glycerol using resting cells of the lactate dehydrogenase‐deficient recombinant *Klebsiella pneumoniae* overexpressing an aldehyde dehydrogenase. Bioresource Technology, 135, 555–563. 10.1016/j.biortech.2012.11.018 23228456

[mbo3956-bib-0027] Kwak, S. , Park, Y. C. , & Seo, J. H. (2013). Biosynthesis of 3‐hydroxypropionic acid from glycerol inrecombinant *Escherichia coli* expressing *Lactobacillus brevis* dhaB and dhaR gene clusters and *E.coli* K‐12aldH. Bioresource Technology, 135, 432–439. 10.1016/j.biortech.2012.11.063 23246300

[mbo3956-bib-0028] Lee, S. H. , Park, S. J. , Park, O. J. , Cho, J. , & Rhee, J. W. (2009). Production of 3‐hydroxypropionic acid from acrylic acid by newly isolated *rhodococcus erythropolis* LG12. Journal of Microbiology & Biotechnology, 19(5), 474–481. 10.4014/jmb.0808.473 19494695

[mbo3956-bib-0029] Li, B. , & Pei, J. (2010). Isolation and Identification of 3‐hyproxypropionic Acid (3HP) Producing Strain. Food Fermentation Industries, 36(4), 28–31.

[mbo3956-bib-0030] Li, S. , Zhu, Z. , Gu, S. , Liu, H. , & Wang, D. (2011). Mutation‐screening in L‐(+)‐lactic acid producing strains by ion implantation. Indian Journal of Microbiology, 51(2), 138–143. 10.1007/s12088-011-0161-y 22654154 PMC3209885

[mbo3956-bib-0031] Li, Y. , Wang, X. , Ge, X. Z. , & Tian, P. F. (2016). High production of 3‐hydroxypropionic acid in *Klebsiella pneumoniae* by systematic optimization of glycerol metabolism. Scientific Reports, 6, 26932 10.1038/srep26932 27230116 PMC4882505

[mbo3956-bib-0032] Lim, H. G. , Noh, M. H. , Jeong, J. H. , Park, S. , & Jung, G. Y. (2016). Optimum rebalancing of the 3‐hydroxypropionic acid productionpathway from glycerol in *Escherichia coli* . ACS Synthetic Biology, 5(11), 1247–1255. 10.1021/acssynbio.5b00303 27056171

[mbo3956-bib-0033] Luo, H. , Zhou, D. F. , Liu, X. H. , Nie, Z. H. , Quiroga‐Sanchez, D. L. , & Chang, Y. H. (2016). Production of 3‐hydroxypropionic acid via the propionyl‐CoA pathway using recombinant *Escherichia coli* strains. PLoS ONE, 11(5), e0156286 10.1371/journal.pone.0156286 27227837 PMC4882031

[mbo3956-bib-0034] Miyoshi, T. , & Harada, T. (1974). Utilization of 2‐butyne‐l,4‐diol by a strain of *Fusarium merismoides* . Journal of Fermentation Technology, 52(6), 196–199.

[mbo3956-bib-0035] Nie, G. , Yang, X. , Liu, H. , Wang, L. I. , Gong, G. , Jin, W. , & Zheng, Z. (2013). N^+^ Ion beam implantation of tannase‐producing *Aspergillus niger* and optimization of its process parameters under submerged fermentation. Annals of Microbiology, 63(1), 279–287. 10.1007/s13213-012-0471-2

[mbo3956-bib-0036] Raj, S. M. , Rathnasingh, C. , Jo, J. E. , & Park, S. (2008). Production of 3‐hydroxypropionic acid from glycerol by a novel recombinant *Escherichia coli* BL21 strain. Process Biochemistry, 43(12), 1440–1446. 10.1016/j.procbio.2008.04.027

[mbo3956-bib-0037] Rathnasingh, C. , Raj, S. M. , Lee, Y. , Catherine, C. , Ashoka, S. , & Park, S. (2012). Production of 3‐hydroxypropionic acid via malonyl‐CoA pathway using recombinant *Escherichia coli* strains. Journal of Biotechnology, 157(4), 633–640. 10.1016/j.jbiotec.2011.06.008 21723339

[mbo3956-bib-0038] Sobolov, M. , & Smiley, K. L. (1960). Metabolism of glycerol by an acrolein‐forming *Lactobacillus* . Journal of Bacteriology, 79(2), 261–266. 10.0000/PMID13832396 13832396 PMC278670

[mbo3956-bib-0039] Song, C. W. , Kim, J. W. , Cho, I. J. , & Lee, S. Y. (2016). Metabolic engineering *of Escherichia coli* for the production of 3‐hydroxypropionic acid and malonic acid through β‐alanine route. ACS Synthetic Biology, 5(11), 1256–1263. 10.1021/acssynbio.6b00007 26925526

[mbo3956-bib-0040] Su, M. Y. , Li, Y. , Ge, X. Z. , & Tian, P. F. (2015). 3‐Hydroxypropionaldehyde‐specifc aldehyde dehydrogenase from *Bacillus subtilis* catalyzes 3‐hydroxypropionic acid production in *Klebsiella pneumoniae* . Biotechnology Letters, 37(3), 717–724. 10.1007/s10529-014-1730-z 25409630

[mbo3956-bib-0041] Takamizawa, K. , Horitsu, H. , Ichikawa, T. , Kawai, K. , & Suzuki, T. (1993). β‐Hydroxypropionic acid production by *Byssochlamys* sp. grown on acrylic acid. Applied Microbiology and Biotechnology, 40(2–3), 196–200. 10.1007/BF00170365

[mbo3956-bib-0042] Talarico, T. D. , Casas, I. A. , Chung, T. C. , & Dobrogosz, W. J. (1988). Production and isolation of reuterin, a growth inhibitor produced by *Lactobacillus reuteri* . Antimicrob Agents and Chemotherapy, 32, 1854–1858. 10.1128/AAC.32.12.1854 PMC176032 3245697

[mbo3956-bib-0043] Tang, M. L. , Wang, S. C. , Wang, T. , Zhao, S. G. , Wu, Y. J. , Wu, L. J. , & Yu, Z. L. (2007). Mutational spectrum of the lacI gene in *Escherichia coli* K12 induced by low‐energy ion beam. Mutation Research/Fundamental and Molecular Mechanisms of Mutagenesis, 602(1–2), 163–169. 10.1016/j.mrfmmm.2006.09.001 17049362

[mbo3956-bib-0044] Wang, P. , Zhang, L. , Zheng, Z. , & Wang, L. (2011). Microbial lipid production by co‐fermentation with *Mortierella alpine*obtained by ion beam implantation. Chemical Engineering & Technology, 34(3), 422–428. 10.1002/ceat.201000370

[mbo3956-bib-0045] Wang, T. , Li, W. , Chen, H. , Li, T. , & Zhao, T. (2014). Breeding of *Cordycepsmilitaris*strain with high Selenium enrichment by low‐energy ion beam implantation. Food Science, 35(15), 136–140. 10.7506/spkx1002-6630-201415028

[mbo3956-bib-0046] Xu, T. , Bai, Z. , Wang, L. , & He, B. (2010). Breeding of D(–)‐Lactic acid high producing strain by low‐energy ion implantation and preliminary analysis of related metabolism. Applied Microbiology and Biotechnology, 160(2), 314–321. 10.1007/s12010-008-8274-4 18574566

[mbo3956-bib-0047] Yao, J. M. , Feng, H. Y. , Yu, Z. L. , Yuan, C. L. , Wang, J. I. , Zheng, Z. M. , … Gong, G. H. (2012). Ion‐beam‐mutation breeding of an arachidonic acid biosynthesis microorganism and its industrial fermentation control. Chinese Science Bulletin, 57(11), 883–890. 10.1360/972011-2199

[mbo3956-bib-0048] Yu, L. , Xu, A. , Wang, J. , & Yu, Z. (1999). The application of low energyion implantation in breeding of high yield Vc strains. Acta Laser Biology Sinica, 8(3), 217–222.

[mbo3956-bib-0049] Yu, S. , Yao, P. , Li, J. , Ren, J. , Yuan, J. , Feng, J. , … Zhu, D. (2016). Enzymatic synthesis of 3‐hydroxypropionic acid at high productivity by using free or immobilizedcells of recombinant *Escherichia coli* . Journal of Molecular Catalysis B‐enzymatic, 129, 37–42. 10.1016/j.molcatb.2016.03.011

[mbo3956-bib-0050] Yu, Z. (2000). Ion beam application in genetic modification. IEEE Transactions on Plasma Science, 28(1), 128–132. 10.1109/27.842882

[mbo3956-bib-0052] Zhang, Q. , Gong, J.‐S. , Dong, T.‐T. , Liu, T.‐T. , Li, H. , Dou, W.‐F. , … Xu, Z.‐H. (2017). Nitrile‐hydrolyzing enzyme from *Meyerozyma guilliermondii* and its potential in biosynthesis of 3‐hydroxypropionic acid. Bioprocess and Biosystems Engineering, 40(6), 901–910. 10.1007/s00449-017-1754-6 28285455

[mbo3956-bib-0053] Zheng, Z. , Zhang, M. , Zhang, G. , & Chen, G. (2004). Production of 3‐hydroxydecanoic acid by recombinant *Escherichia coli* HB101 harboring phaG gene. Antonievan Leeuwenhoek, 85(2), 93–101. 10.1023/B:ANTO.0000020275.23140.ca 15031653

[mbo3956-bib-0054] Zhou, S. F. , Catherine, C. , Rathnasingh, C. , Somasundar, A. , & Park, S. (2013). Production of 3‐hydroxypropionic acid from glycerolby recombinant Pseudomonas denitrificans. Biotechnology and Bioengineering, 110(12), 3177–3187. 10.1002/bit.24980 23775313

[mbo3956-bib-0055] Zhu, G. , & Wang, Z. (1994). Laboratory technical handbook of industry microorganisms (p. 391). Beijing, China: Light Industry Pressing Company of China.

